# Association between cervical length and gestational age at birth in singleton pregnancies: a multicentric prospective cohort study in the Brazilian population

**DOI:** 10.1186/s12978-022-01557-w

**Published:** 2023-03-22

**Authors:** Thais Valéria Silva, Anderson Borovac-Pinheiro, José Guilherme Cecatti, Ben Willem Mol, Fabricio Silva Costa, Marcelo Santucci França, Renato Teixeira Souza, Roland Devlieger, Renato Passini, Rodolfo Carvalho Pacagnella, The P5 working group, Allan R Hatanaka, Amanda Dantas, Antonio Fernandes Moron, Carlos Augusto Santos Menezes, Cláudio Sérgio Medeiros Paiva, Cristhiane B Marques, Cynara Maria Pereira, Daniela dos Santos Lopes Homenko, Djacyr Magna Cabral Paiva, Elaine Christine Dantas Moisés, Enoch Quinderé Sá Barreto, Felipe Soares, Fernando Maia Peixoto-Filho, Francisco Edson de Lucena Feitosa, Francisco Herlanio Costa Carvalho, Jessica Scremin Boechem, João Renato Benini-Junior, José Airton Oliveira Lima, Juliana P. Argenton, Kaline F Marquart, Karayna Gil Fernandes, Kleber Cursino Andrade, Leila Katz, Maíra Rossmann Machado, Marcelo L Nomura, Marcelo Marques Souza Lima, Marcos Nakamura-Pereira, Maria Julia Miele, Maria Laura Costa, Mário Correia Dias, Nathalia Ellovitch, Nelson Sass, Rodrigo Pauperio Soares Camargo, Sabrina de Oliveira Silva Savazoni, Samira El Maerrawi Tebecherane Haddad, Sérgio Martins-Costa, Silvana F Bento, Silvana Maria Quintana, Stéphanno Gomes Pereira Sarmento, Tatiana F Fanton, Thaísa Bortoletto Guedes, Valter Lacerda de Andrade Junior

**Affiliations:** 1 Department of Obstetrics and Gynecology, School of Medicine, University of Campinas, Campinas, Brazil; 2 CISAM Maternity Hospital, University of Pernambuco, Recife, Brazil; 3 Department of Obstetrics and Gynaecology, Monash University, Victoria, Australia; 4 Aberdeen Centre for Women’s Health Research, University of Aberdeen, Aberdeen, UK; 5 Maternal Fetal Medicine Unit, Gold Coast University Hospital and School of Medicine, Griffith University, Southport, Australia; 6 Screening and Prevention of Preterm Birth Sector, Fetal Medicine Discipline, Obstetrics Department, Escola Paulista de Medicina, Federal University of Sao Paulo, Sao Paulo, Brazil; 7 Department of Obstetrics and Gynaecology, University Hospitals KU Leuven, Louvain, Belgium

**Keywords:** Cervical length, Number needed to screen, Preterm birth, Short cervix, Pregnancy

## Abstract

**Background:**

Short cervical length measured during the second trimester of pregnancy is an important risk factor for spontaneous preterm birth (sPTB). The aim of this study is to identify the association between mid-pregnancy cervical length (CL) and gestational age at birth in asymptomatic singleton pregnant women.

**Methods:**

This is a prospective cohort study involving singleton pregnant women who participated in the screening phase of a Brazilian multicenter randomized controlled trial (P5 trial) between July 2015 and March 2019. Transvaginal ultrasound to measure CL was performed from 18 to 22 + 6 weeks. Women with CL ≤ 30 mm received vaginal progesterone (200 mg/day) until 36 weeks’ gestation. For this analysis we considered all women with CL ≤ 30 mm receiving progesterone and a random selection of women with CL > 30 mm, keeping the populational distribution of CL. We obtained prognostic effectiveness data (area under receive operating characteristic curve (AUC), sensitivity and specificity and estimated Kaplan–Meier curves for preterm birth using different CL cutoff points.

**Results:**

We report on 3139 women and identified a negative association between cervical length and sPTB. CL ≤ 25 mm was associated with sPTB < 28, sPTB < 34 and sPTB < 37 weeks, whereas a CL 25–30 mm was directly associated with late sPTB. CL by transvaginal ultrasound presented an AUC of 0.82 to predict sPTB < 28 weeks and 0.67 for sPTB < 34 weeks. Almost half of the sPTB occurred in nulliparous women and CL ≤ 30 mm was associated with sPTB at < 37 weeks (OR = 7.84; 95%CI = 5.5–11.1). The number needed to screen to detect one sPTB < 34 weeks in women with CL ≤ 25 mm is 121 and we estimated that 248 screening tests are necessary to prevent one sPTB < 34 weeks using progesterone prophylaxis.

**Conclusions:**

CL measured by transvaginal ultrasound should be used to predict sPTB < 34 weeks. Women with CL ≤ 30 mm are at increased risk for late sPTB.

**Supplementary Information:**

The online version contains supplementary material available at 10.1186/s12978-022-01557-w.

## Introduction

Prematurity is the leading cause of neonatal morbidity and mortality [1], with severe emotional sequelae and high economic costs. Nowadays, the Preterm Birth (PTB) rate is 10.6% worldwide and 11.2% in Brazil, higher than suggested by the World Health Organization [2, 3]. There are 15 million PTBs each year and the burden is directly associated with gestational age at birth.

To prevent PTB bad outcomes, studies have focused on identifiable risk factors such as having a short cervix. Early uterine cervical shortening in the second trimester is an important risk factor for prematurity [4] and is associated with spontaneous preterm birth (sPTB). Thus, cervical length (CL) measurement during the second trimester could be used as a tool to identify women at risk of premature delivery [5]. Transvaginal ultrasound (TVU) performed during the second trimester can evaluate cervical shortening before labor and then a universal screening test has been proposed [6]. Nevertheless, the CL cutoff point related to PTB is still in debate. Most studies consider CL ≤ 25 mm as a risk factor, whereas others consider higher or lower cutoff points [7–9].

Predicting PTB among pregnant women is the key to preventive interventions [10]. Thus, the aim of this study is to identify the association between CL at 18–22(+ 6) weeks of pregnancy and gestational age at birth in asymptomatic Brazilian women with singleton pregnancy and to assess the performance of TVU as a screening test to predict PTB.

## Methods

This is a prospective multicenter cohort study involving singleton pregnant women screened during a multicenter randomized controlled trial entitled “Pessary plus Progesterone for Preventing Preterm Birth” (P5 trial; Registration no. RBR-3t8prz, approved by the Brazilian National Review Board/CONEP—number 1.055.555) [11]. The P5 trial was conducted by the University of Campinas (UNICAMP) and involved 17 centers in nine states of Brazil from July 2015 to March 2019. Women between 18 and 22(+ 6/7) gestational weeks were invited to participate in the P5 screening phase. A consent form was signed and TVU was performed to measure the CL.

The standard technique followed the P5 study protocol and the Fetal Medicine Foundation orientation for CL measurement. Briefly, with the woman in dorsal lithotomy position and empty bladder, a TVU probe was introduced inside the vagina until the anterior fornix avoiding pressure. A sagittal view of the cervix, including the edge, identified the internal and external ostium. Calipers were used to measure the linear distance (in mm) between the external and internal ostium. Funneling and Sludge were described. All data from the screening phase were included in the online database Gsdoctor. Every participating center stored their ultrasound images with the CL measurements to confirm that all centers were correctly applying the TVU technique.

All women with a CL ≤ 30 mm who did not have exclusion criteria and who accepted to participate in the trial were randomized into two groups: 200 mg/day vaginal progesterone or 200 mg/day vaginal progesterone + cervical pessary. Randomized women have delivery information in the P5 database. Women with CL > 30 mm had their childbirth and postnatal information collected from hospital medical registers and added to the P5 database.

The sample for this analysis considered all women with CL ≤ 30 mm receiving only progesterone and a random selection of women with CL > 30 mm, keeping the populational distribution of cervical length. Women using cervical pessary were excluded since we did not have clear information of how it could influence the gestational age at birth and this treatment is not routine for preventing PTB. Considering that progesterone is an established evidence-based treatment for preventing PTB and women are encouraged to use it if they have a short CL identified in the mid-trimester, we included the P5 trial progesterone group in our cohort sample. The P5 trial total sample screened 13.7% women with CL ≤ 30 mm and 86.3% of CL > 30 mm. To maintain the same CL distribution, we projected the progesterone group to correspond to 13.7% of CL ≤ 30 mm for our analysis. To complete our final sample and reach the complementary 86.3% of CL > 30 mm, we selected singleton women with CL > 30 mm using a random model. We excluded women who had received a cervical pessary, multiple gestations and those with incomplete gestational outcome data. We kept very similar baseline characteristics percentages found in the total of singleton pregnant that participated in the P5 trial screening, maintaining homogeneity and avoiding any possible selection bias (Additional file 1). The primary outcome was PTB at < 37 weeks’ gestation and secondary outcomes were sPTB at < 37, < 34, < 32 and < 28 weeks’ gestation.

Descriptive statistical analysis was performed for demographic characteristics, expressed as means and percentages. Logistic regression was used to estimate odds ratios for baseline characteristics, gestational age and CL at measurement. A multivariate logistic regression analysis was performed to estimate adjusted odds ratio for different gestational ages.

For our primary outcome, receiver operating characteristic (ROC) curve analysis was performed to identify the most effective cutoff point to predict a PTB (< 37 weeks). Our secondary outcomes were ROC curve analysis to identify the most effective cutoff points to predict sPTB at different gestational ages (< 37, < 34, < 32 and < 28 weeks). Kaplan-Meyer survival curves were used to analyze time to delivery, considering CL intervals (≤ 10 mm, 10–15 mm, 15–20 mm, 20–25 mm, 25–30 mm, 30–35 mm, 35–40 mm and > 40 mm). We calculated the number needed to screen (NNS) to detect one true positive sPTB < 34 in women with CL ≤ 25 mm. Considering a recent individual patient data (IPD)-metanalysis that included randomized clinical trials involving women with CL ≤ 25 mm treated with vaginal progesterone, the number needed to treat (NNT) with vaginal progesterone to prevent one sPTB < 34 weeks is 18 [12]. Therefore, we estimated the number of TVU necessary to identify 18 women with CL ≤ 25 mm. *P* < 0.05 was considered as statistically significant. All statistical analyses were performed using R version 3.6.2 software.

## Results

The P5 trial screened 8168 women, of whom 7857 were singleton and 1081 had CL ≤ 30 mm. In a CL distribution curve including only singleton pregnancies, 1081 women corresponds to 13.7% of total. For this study, we excluded 310 twins, 14 women without CL data and 3 women in progesterone group without gestational age at birth. We included 430 singleton women with CL ≤ 30 mm randomized to progesterone alone and we projected this group to correspond to 13.7% of CL ≤ 30 mm for our analysis. To complete our final sample and reach the complementary 86.3% of CL > 30 mm, we randomly selected 2709 singleton women with CL > 30 mm, comprising a total of 3139 women (Additional file 5).

Among women with CL ≤ 30 mm receiving progesterone, compliance was 82%. Regarding obstetric history, 46.2% (1449) of our sample were nulliparous, 10.1% (318) had at least one previous PTB and 24.4% had a previous miscarriage. The prevalence of PTB at < 37 weeks was 14.43%: sPTB at < 37 weeks was found in 7.1% (223/3139); and sPTB at < 37 weeks in women with CL ≤ 30 mm receiving progesterone was 16.7% (72/430). Of all 223 women who had a sPTB, 32.3% (72/223) had a CL ≤ 30 mm. Sociodemographic information is listed in Table 1.

**Table 1:** Sociodemographic and baseline characteristics x gestational age at birth

Characteristics	Overall PTB < 37 (n = 453)		≥ 37w (n = 2686)		OR (95%CI)	Spontaneous (sPTB) < 37 (n = 223)		≥ 37w (n = 2686)		OR (95%CI)
	n or Mean	% or ± SD	n or Mean	% or ± SD		n or Mean	% or ± SD	n or Mean	% or ± SD	
Maternal age at measurement (years)	28.7	± 7	27.8	± 7		27.4	± 6.9	27.8	± 7	
≤ 19	56	12.4	405	15.1		36	16.2	405	15.1	
20– ≤ 34	307	67.9	1794	67.1	1.24 (0.92–1.69)	152	68.5	1794	67.1	0.95 (0.66–1.41)
> 35	89	19.7	476	17.8	1.35 (0.95–1.95)	34	15.3	476	17.8	0.80 (0.49–1.31)
Body-mass index (kg/m^2^)										
≤ 18.5	16	3.5	52	1.9	1.95 (1.05–3.43)	10	4.5	52	1.9	2.07 (0.96–4.06)
18.5–25	148	32.7	937	34.9		87	39.0	937	34.9	
25–30	157	34.7	913	34.0	1.09 (0.85–1.39)	72	32.3	913	34.0	0.85 (0.61–1.17)
> 30	132	29.1	784	29.2	1.07 (0.83–1.37)	54	24.2	784	29.2	0.74 (0.52–1.05)
Ethnic origin (self-reported)										
Non-white	289	63.8	1680	62.5		143	64.1	1680	62.5	
White	164	36.2	1006	37.5	0.95 (0.77–1.16)	80	35.9	1006	37.5	0.93 (0.70–1.24)
Schooling										
Preschool, elementary	116	25.8	711	26.6		55	24.9	711	26.6	
Middle school	275	61.2	1666	62.3	1.01 (0.80–1.28)	140	63.3	1666	62.3	1.09 (0.79–1.51)
High school and higher education	58	12.9	298	11.1	1.19 (0.84–1.67)	26	11.8	298	11.1	1.13 (0.68–1.81)
Comorbidities										
No comorbidities	285	62.9	1992	74.2		163	73.1	1992	74.2	
Hypertension	47	10.4	153	5.7	2.15 (1.50–3.02)	8	3.6	153	5.7	0.64 (0.28–1.24)
Endocrinopathies^a^	63	13.9	254	9.5	1.73 (1.27–2.33)	28	12.6	254	9.5	1.35 (0.87–2.02)
Cardiovascular disease	2	0.4	18	0. 7	0.78 (0.12–2.71)	1	0.45	18	0.7	0.68 (0.04–3.32)
Others^b^	56	12.4	269	10.0	1.46 (1.06–1.98)	23	10.3	269	10.0	1.04 (0.65–1.61)
Previous conization(yes)	9	1.9	36	1.3	1.33 (0.57–2.73)	3	1.3	36	1.3	1.00 (0.24–2.81)
Uterine anomaly (yes)	9	1.9	36	1.3	1.50 (0.67–2.99)	3	1.3	36	1.3	1.00 (0.24–2.81)
Obstetrical history										
Nulliparous	205	45.4	1244	46.3		109	48.9	1244	46.3	
Parous with no previous PTB	154	34.1	1217	45.3	0.77 (0.61–0.96)	69	30.9	1217	45.3	0.65 (0.47–0.88)
Parous with at least one previous PTB	93	20.6	225	8.4	2.51 (1.88–3.32)	45	20.2	225	8.4	2.28 (1.56–3.30)
Previous miscarriage (yes)	138	30.5	629	23.4	1.43 (1.15–1.78)	69	30.9	629	23.4	1.47 (1.08–1.97)

Characteristics	Spontaneous (sPTB) < 34 (n = 78)		≥ 34w (n = 2976)		OR (95%CI)
	n or Mean	% or ± SD	n or Mean	% or ± SD	
Maternal age at measurement (years)	27.1	± 7.2	27.9	6.9	
≤ 19	15	19.2	437	14.7	
20– ≤ 34	49	62.8	2002	67.5	0.71 (0.41–1.33)
> 35	14	17.9	525	17.7	0.78 (0.37–1.63)
Body-mass index (kg/m^2^)					
≤ 18.5	6	7.7	62	2.1	3.01 (1.10–6.98)
18.5–25	33	42.3	1026	34.5	
25–30	23	29.5	1021	34.3	0.70 (0.40–1.19)
> 30	16	20.5	867	29.1	0.57 (0.31–1.03)
Ethnic origin (self-reported)					
Non-white	46	59.0	1869	62.8	
White	32	41.0	1107	37.2	1.17 (0.74–1.85)
Schooling					
Preschool, elementary	18	23.1	784	26.5	
Middle school	50	64.1	1842	62.2	1.18 (0.70–2.09)
High school and higher education	10	12.8	335	11.3	1.30 (0.57–2.79)
Comorbidities					
No comorbidities	50	64.1	2180	73.3	
Hypertension	3	3.8	181	6.1	0.72 (0.17–1.99)
Endocrinopathies^a^	12	15.4	294	9.9	1.78 (0.90–3.27)
Cardiovascular disease	0	0.0	20	0.7	–
Others^b^	13	16.7	301	10.1	1.88 (0.97–3.40)
Previous conization(yes)	2	2.6	41	1.4	1.88 (0.30–6.28)
Uterine anomaly (yes)	1	1.3	38	1.3	1.00 (0.06–4.72)
Obstetrical history					
Nulliparous	44	56.4	1363	45.8	
Parous with no previous PTB	17	21.8	1330	44.7	0.40 (0.22–0.68)
Parous with at least one previous PTB	17	21.8	282	9.5	1.87 (1.02–3.26)
Previous miscarriage (yes)	27	34.6	709	23.8	1.69 (1.04–2.70)

Data are number (%) or mean (± SD)*.* OR values in bold mean that they are significant at a *P-*value < 0.05. BMI was calculated at CL measurement

^a^Diabetes Mellitus, gestational diabetes, thyroidopathy

^b^Asthma, autoimmune diseases, anemia, obesity, hepatitis

Logistic univariate regression analysis for PTB at < 37 weeks identified the following risk factors: low body mass index (BMI ≤ 18.5) (OR = 1.95, 95%CI = 1.05–3.43,); hypertension (OR 2.15, 1.5–3.02); endocrinopathies (OR = 1.73, 1.27–2.33); previous PTB (OR = 2.51, 1.88–3.32); previous miscarriage (OR = 1.43, 1.15–1.78); cervical length ≤ 30 mm (CL 25– ≤ 30 mm OR 2.10, 1.47–2.95; CL 20–25 mm OR 2.55, 1.71–3.72; CL 15–20 mm OR 3.33, 1.74–6.11; CL 10–15 mm OR = 6.40, 2.53–5.99, and CL ≤ 10 mm OR 11.17, 4.37–30.55); funneling at measurement (OR = 5.03, 3.36–7.49); and sludge at measurement (OR = 3.50, 2.24–5.39). Considering only sPTB at < 37 weeks, these factors presented an even higher association except for comorbidities and low BMI. A comparison between sPTB at < 34 weeks and ≥ 34 weeks illustrates that there is a robust association among risk factors and sPTB < 34 weeks, highlighting CL ≤ 10 mm (OR 44.9, 15.45–125.87) and 10–15 mm (OR13.32, 2.98–43.09), funneling at measurement (OR 10.22, 5.57–17.95) and sludge at measurement (OR = 5.61, 2.63–10.86) (Table 2).

**Table 2:** Cervical length measurement and gestational age at birth

		Overall PTB < 37		≥ 37w		OR (95%CI)	Spontaneous (sPTB) < 37			≥ 37w		OR (95%CI)	Spontaneous (sPTB) < 34		≥ 34w		OR (95%CI)
		n or Mean	% or ± SD	n or Mean	% or ± SD		n or Mean	% or ± SD	n or Mean		% or ± SD		n or Mean	% or ± SD	n or Mean	% or ± SD	
GA at measurement (days)		145.9	± 8.8	146	± 8.8				146		± 8.8		144.6	± 8.6	146.0	± 8.8	
CL at measurement (mm)																	
≤ 10 mm		11	2.4	7	0.3	11.17(4.37–30.55)	8	3.6	7		0.3	17.98 (6.37–51.90)	7	8.9	9	(0.3	44.9(15.45–125.87)
10–≤ 15 mm		9	1.9	10	0.4	6.4 (2.53–15.99)	6	2.7	10		0.4	9.44(3.17–25.76)	3	3.8	13	0.4	13.32 (2.98–43.09)
15–≤ 20 mm		15	3.3	32	1.2	3.33 (1.74–6.11)	9	4.0	32		1.2	4.42 (1.96–9.06)	6	7.7	40	1.3	8.66 (3.17–20.09)
20–≤ 25 mm		38	8.4	106	3.9	2.55 (1.71–3.72)	20	8.9	106		3.9	2.97 (1.75–4.82)	10	12.8	124	4.2	4.66 (2.17–9.09)
25–≤ 30 mm		46	10.2	156	5.8	2.10 (1.47–2.95)	29	13.0	156		5.8	2.92 (1.87–4.43)	7	8.9	192	6.4	2.10 (0.86–4.44)
> 30 mm		334	73.7	2375	88.4		151	67.7	2375		88.4		45	57.7	2598	87.3	
Funneling at measurement (yes)		46	10.2	59	2.2	5.03 (3.36–7.49)	30	13.5	59		2.2	6.92(4.31–10.92)	17	21.8	79	2.6	10.22 (5.57–17.95)
Sludge at measurement (yes)		33	7.3	59	2.2	3.50 (2.24–5.39)	18	8.1	59		2.2	3.91 (2.20–6.62)	10	12.8	76	2.5	5.61 (2.63–10.86)

Data are number (%) or mean (± SD)*.* OR values in bold mean that they are significant at a *P-*value < 0.05

*GA* gestational age, *CL *cervical length

A multivariate logistic regression analysis also identified an association between CL ≤ 30 mm and PTB (CL 25– ≤ 30 mm ORa 1.80, 1.23–2.63; CL 20–25 mm ORa 1.93, 1.22–3.06; CL 10–20 mm ORa 3.04, 1.54–5.71, and CL ≤ 10 mm ORa 3.82, 1.12–13.06). The ORa for cervical length < 30 mm increased when considered only sPTB < 37 (CL 25– ≤ 30 mm ORa 2.2, 1.35–3.57; CL 20–25 mm ORa 2.07, 1.14–3.76; CL 10–20 mm ORa 4.59, 2.12–9.94, and CL ≤ 10 mm ORa 6.71, 1.79–25.27). For sPTB < 34, there was an association with CL ≤ 25 mm (Additional file 2). We also performed a multivariate analysis for cervical length and PTB < 37, sPTB < 37 and sPTB < 34 weeks with adjusted odds ratios for BMI, comorbidities, obstetrical history, funneling and sludge and the association between CL < 30 mm and PTB and sPTB < 37 was also significant. Again, moderate sPTB (sPTB < 34) where associated with CL ≤ 25 mm (Additional file 3).

**Fig. 1:**
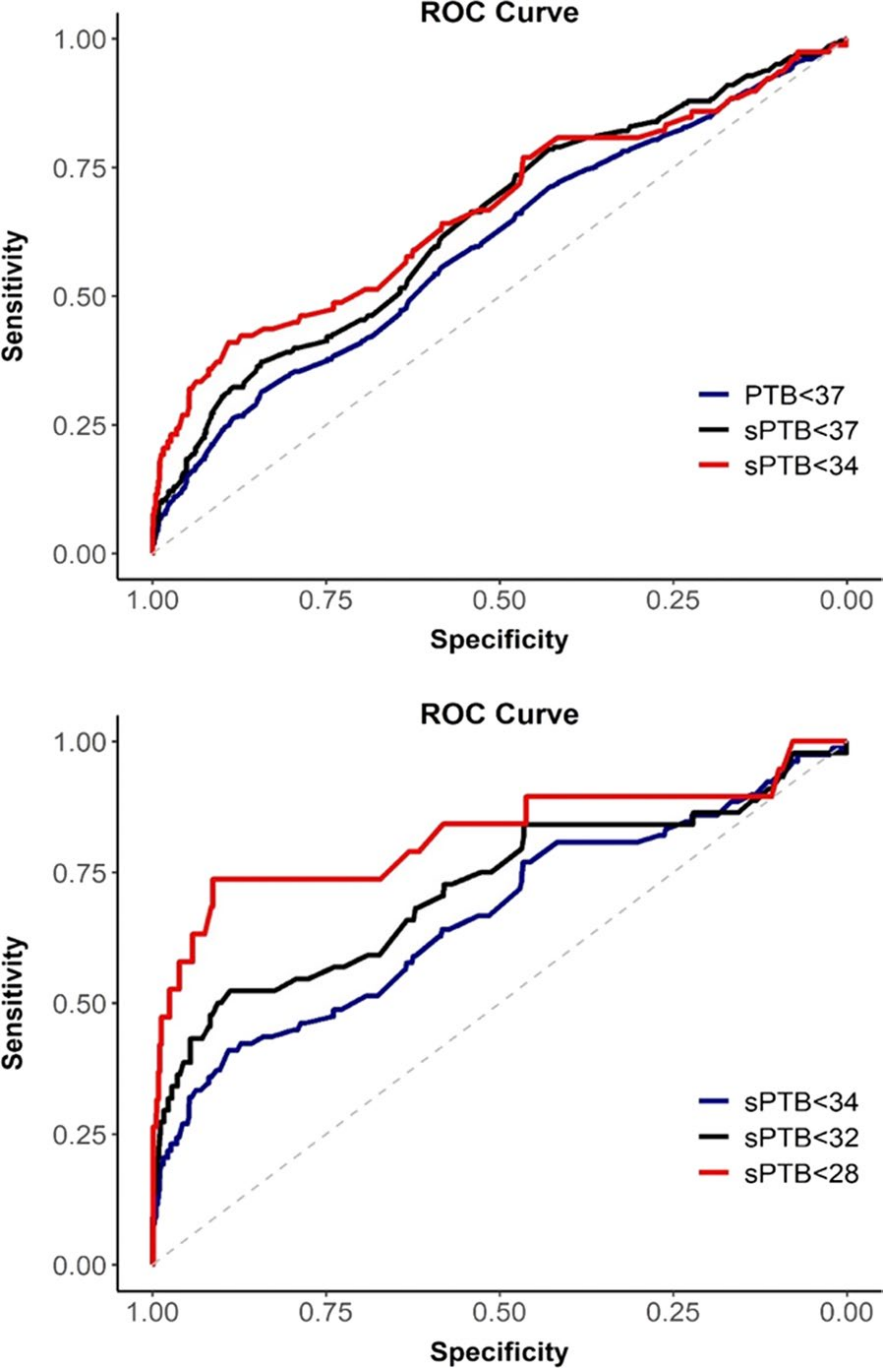
ROC curve analysis of PTB and sPTB at different gestational ages

We identified an inverse association between CL and sPTB at < 37 weeks (OR = 7.84, 5.5–11.1). The ROC curve analysis to predict PTB at < 37 weeks and sPTB at < 37 weeks showed low performance, with area under the curve (AUC) of 0.598 (0.57–0.63) and 0.643 (0.60–0.68), respectively. For sPTB at < 34 weeks and sPTB at < 32 weeks the ROC curve presented a moderate performance with AUC of 0.665 (0.59–0.74) and 0.718 (0.62–0.81), respectively; and for sPTB at < 28 weeks the ROC curve demonstrated good performance, with AUC of 0.820 (0.63–0.95) (Additional file 4; Fig. 1).

The best cutoff point to predict PTB at < 37 weeks was 31.75 mm, with 31.3% sensitivity and 84.4% specificity. To predict sPTB at < 37 weeks the best cutoff point was 31.75 mm, with 37.2% sensitivity and 84.3% specificity. TVU provided good prognostic results combining: AUC (0.82), high sensitivity (73.7%) and acceptable specificity (91.3%) rates for sPTB at < 28 weeks’ gestation (Additional file 4: Table S4). The best cutoff points to predict sPTB at < 34, < 32 and < 28 weeks were 28.05, 28.05 and 26.55 mm, respectively.

**Fig. 2:**
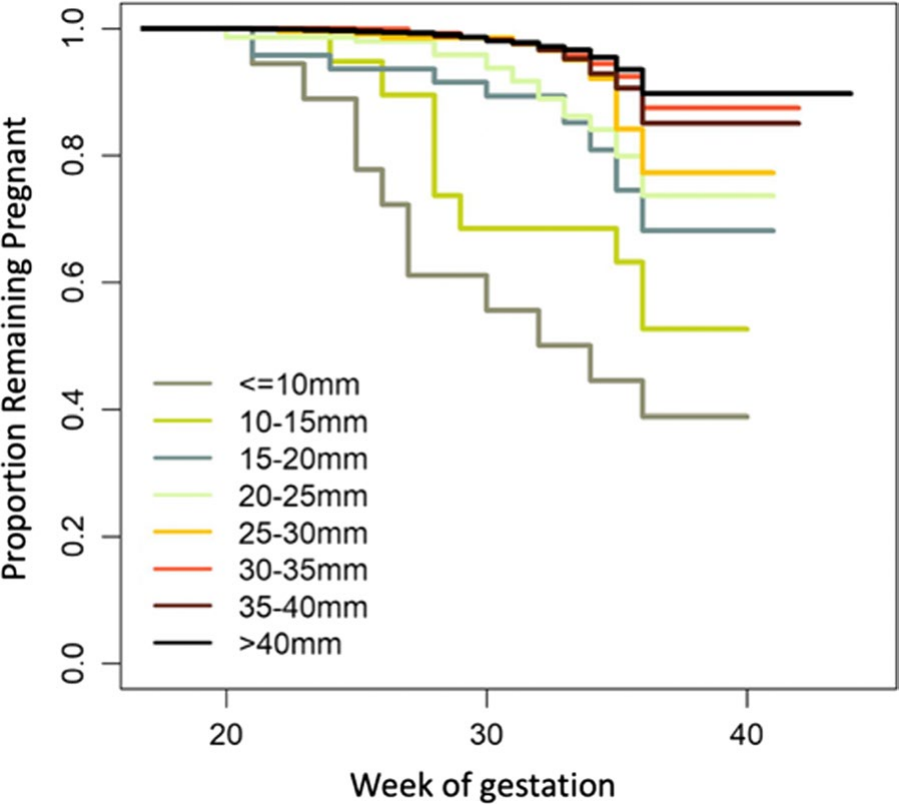
Kaplan-Meyer survival analysis for PTB considering different ranges of CL

Kaplan-Meyer survival analysis demonstrated an association between extremely severe, severe, moderate and late PTB and CL ≤ 25 mm, and an association between CL of 25–30 mm and late PTB (*p* < 0.001) (Fig. 2). For sPTB and CL ≤ 25 mm, see Additional file 6.

The number needed to screen (NNS) to detect one true positive sPTB < 34 weeks in women with CL ≤ 25 mm is 121. To prevent one sPTB < 34 weeks among women with CL ≤ 25 mm, the number needed to treat (NNT) with vaginal progesterone prophylaxis is 18^12^. Assuming that all women with CL ≤ 25 mm are treated with vaginal progesterone, we estimated that the number of TVU necessary to identify 18 women with CL ≤ 25 mm and prevent one sPTB < 34 weeks is 248.

## Discussion

Our study identified a negative association between CL measured during the second trimester of pregnancy and the rate of sPTB. CL ≤ 31.7 mm is an important risk factor for PTB at ≤ 37 weeks and CL ≤ 25 mm is associated with extremely severe, severe, moderate and late PTB whereas CL of 25–30 mm is associated with late PTB. This study also confirms previous observational studies that found low BMI, previous miscarriage, previous PTB, CL ≤ 30 mm, funneling and sludge as predictors for PTB [13–15].

The most relevant risk factor for PTB in a singleton pregnancy is a previous history of PTB; however, in nulliparous women this does not apply. We had almost half of the sPTB in nulliparous women and TVU is an important mean to identify nulliparous women at risk of PTB. In those women, except for BMI, the other important risk factors are directly connected to the second trimester TVU results. Thus, considering the higher incidence of sPTB in Brazil and globally [16], TVU is an important tool to routinely identify these women.

As a screening test for PTB, TVU did not present good performance to predict PTB at < 37 weeks. This result agrees with previous studies that did not find high sensitivity or acceptable specificity to consider TVU as a screening test to predict late PTB [17, 18]. Nevertheless, we can consider that TVU has a moderate prognostic performance to predict sPTB at < 34 weeks and, moreover, has a good performance for predicting sPTB at < 28 weeks, with a high sensitivity and acceptable specificity. The extremely severe and severe PTB correspond to only 5% of all premature deliveries but are responsible for most deaths associated with PTB [3].

There is an inverse correlation between long-term morbidity and adverse neurodevelopmental outcomes with gestational age at birth, which incurs higher medical costs and extrapolates this health problem to the economic sphere, generating a huge financial impact on the health system. The suggested NNS to identify a woman under real risk for an early preterm birth is very acceptable for a screening test. Thus, offering TVU as a screening test for women at risk of moderate and extreme sPTB would increase the reaching of optimal timing for antenatal corticosteroid administration [19] and allow preventive treatments for reducing sPTB as progesterone, cervical pessary or cerclage [8, 20].

Recently, a multicenter Swedish cohort study involving 11,465 asymptomatic singleton pregnant women found that TVU ability to predict sPTB at < 37 weeks was poor: AUC of 0.63 (0.59–0.67) for measurement at 21–23 (+ 6) weeks with best cutoff point 35 mm; and the number needed to screen (NNS) to detect one true positive test result for sPTB at < 34 weeks considering CL ≤ 25 mm was 524. TVU demonstrated good performance (AUC > 0.75) for predicting sPTB at < 31 weeks’ gestation [21]. Despite the considerable differences between our population and theirs, including the fact that our women used progesterone if CL ≤ 30 mm and the difference between sPTB rates (7.1% our study versus 3.6% Swedish study), both studies illustrate that 25 mm does not seem to be the best cutoff point to identify women at PTB risk; moreover, TVU has moderate or good accuracy when different gestational ages are considered in both analyses. In addition, our NNS to identify one true positive sPTB < 34 weeks when women with CL ≤ 25 mm is considerably lower than previous studies that considered populations with lower PTB rate [21, 22], what is an alert to correctly define the applicability and cost-utility of TVU-CL measurement as a screening test for PTB in different countries.

The main strength of this study is that we have a considerably large sample of Brazilian women from 17 centers in three regions, thus covering possible internal population differences. In Brazil, previous TVU performance analyses to predict PTB were from single-center studies [18, 23] with smaller samples. All cervical measurements were performed by expert medical sonographers in tertiary reference centers, along with checking of the ultrasound images to correct and reinforce the pattern technique. We analyzed TVU using different accuracy tests, different cutoff points and specific PTB subgroups for gestational age.

The vaginal progesterone used for women with CL ≤ 30 mm is a limitation in our study because progesterone reduces the occurrence of PTB. Nevertheless, in our prenatal clinical assistance, women with CL ≤ 25 mm are encouraged to use progesterone, so maintaining this intervention in our sample allows the possibility to pragmatically infer the results to medical practice. Unfortunately, we cannot identify if progesterone has caused any reduction in PTB between women with CL 25– ≤ 30 mm, which could have underestimated PTB incidence in this subgroup. Another limitation is that some participating centers did not perform universal TVU screening, which could introduce some selection bias in our sample and the tendency to have a shorter CL. However, the mean CL identified was very similar to other previous Brazilian studies [16, 24, 25].

Women with CL ≤ 25 mm had a significant association with sPTB < 34 weeks, which is an important clinical goal for preterm birth. Additionally, we found that the best cutoff points for all gestational ages outcomes (< 37, < 34, < 32 and < 28 weeks) are over 25 mm. Considering the feasibility to perform CL measurement following a standard technique and the capability to detect almost one third of all sPTB < 37 weeks, we suggest to use CL ≤ 30 mm as the cutoff for cervical length to identify women at risk of sPTB. This is easier to remember and is very similar to the best cutoff point identified in our study. Thus, women with CL ≤ 30 mm should be recognized as at higher risk for PTB and those with CL ≤ 25 mm should be recognized and treated properly to reduce sPTB < 34 weeks.

It is important to highlight that although women with CL ≤ 30 mm are at higher risk for PTB, effective treatment for preventing PTB in women with 25–30 mm CL are not available [26]. These women should not be treated with progesterone, cervical pessaries, or cerclage because these treatments did not show clear benefits in reducing sPTB but should, however, receive a close antenatal care follow-up.

Considering the cutoff point where vaginal progesterone has demonstrated efficacy (25 mm), the NNS of 248 to detect 18 women with CL ≤ 25 mm is an acceptable number, which suggests the feasibility of implementing TVU for pregnant women in mid-trimester in settings like Brazil.

As most PTBs worldwide are concentrated in low- and middle-income countries, this analysis is important to describe specific results for our population and stimulate new studies in other similar settings focused on strategies to reduce PTB. In such countries, where economical resources are considerably limited, it is important to define with precision the best strategies to reduce costs while improving health care. Nowadays, the national antenatal care for Brazil has not adopted routine TVU at mid-trimester screening based on studies developed in high-income countries with lower rates of sPTB. The NNS estimated in our study creates an opportunity to review the Brazilian and other countries’ protocols to deal with the PTB prevention. The estimated NNS is considered low and acceptable and should underpin the implementation of the TVU as a mid-trimester screening test.

## Conclusions

Cervical length CL ≤ 25 mm measured by transvaginal ultrasound in the second trimester should be used to predict spontaneous preterm birth < 34 weeks of gestation. The NNS is considered low and acceptable and should underpin the implementation of the TVU as a mid-trimester screening test. Women with CL ≤ 30 mm can also be considered at higher risk for PTB in the Brazilian population.

## Supplementary Information

**Additional file 1:** Comparisonof socio-demographics and obstetrics characteristics between the cohort and P5trial screening phase (only singleton pregnancies).

**Additional file 2**: Multivariatelogistic regression analysis for total and sPTB at different gestational ages.

**Additional file 3**: Cervicallength x PTB with adjusted OR for BMI, comorbidities, obstetrical history,funneling and sludge (Table S3.1, S3.2 and S3.3).

**Additional file 4**: TVUmeasurement of CL performance for predicting PTB.

**Additional file 5**: Women enrolment flowchart.

**Additional file 6**: Kaplan-Meyer survival analysis for sPTB considering different ranges ofCL.

## Data Availability

The datasets used and/or analysed during the current study are available from the corresponding author on reasonable request.

## Abbreviations

CL: Cervical length
TVU: Transvaginal ultrasound
AUC: Area under the curve
OR: Odds ratio
PTB: Preterm birth
sPTB: Spontaneous preterm birth
ROC: Receiver operating characteristic curve
NNS: Number needed to screen
NNT: Number needed to treat
IPD: Individual patient data
BMI: Body mass index
GA: Gestational age
