# Supplementary material for: Association between cervical length and gestational age at birth in singleton pregnancies: a multicentric prospective cohort study in the Brazilian population
Source: Reprod Health. 2023 Mar 22;20:47. doi: 10.1186/s12978-022-01557-w (PMC10035243; doi:10.1186/s12978-022-01557-w)
Supplement: Supplementary file 1 — Additional file 1: Comparisonof socio-demographics and obstetrics characteristics between the cohort and P5trial screening phase (only singleton pregnancies). [file 12978_2022_1557_MOESM1_ESM.docx]

Additional file 1- Comparison of socio-demographics and obstetrics characteristics between the cohort and P5 trial screening phase (only singleton pregnancies)

| **Baseline characteristics** | | **Cohort** | |  | **P5 trial** | |
| --- | --- | --- | --- | --- | --- | --- |
|  |  | **n (3139)** | **Freq %** |  | **n (7844)** | **Freq %** |
| Maternal age at measurement (years) | |  |  | 27.8 |  |  |
|  | ≤19 | 461 | 14,7% | 405 | 924 | 11,8% |
|  | 20- ≤34 | 2101 | 66,9% | 1794 | 5425 | 69,2% |
|  | >35 | 565 | 18,0% | 476 | 1495 | 19,0% |
| Body-mass index (kg/m^2^) | | 3139 |  |  |  |  |
|  | ≤18.5 | 68 | 2,2% | 52 | 178 | 2,3% |
|  | 18.5-25 | 1085 | 34,6% | 937 | 2814 | 35,9% |
|  | 25-30 | 1070 | 34,1% | 913 | 2630 | 33,5% |
|  | >30 | 916 | 29,2% | 784 | 2222 | 29,2% |
| Ethnic origin (self-reported) | |  |  |  |  |  |
|  | Non-white | 1969 | 62,7% | 1680 | 4901 | 62,5% |
|  | White | 1170 | 37,3% | 1006 | 2943 | 37,5% |
| Schooling | |  | 0,0% |  |  |  |
|  | Preschool, Elementary | 827 | 26,3% | 711 | 1889 | 24,1% |
|  | Middle School | 1941 | 61,8% | 1666 |  |  |
|  | High School and Higher Education | 356 | 11,3% | 298 | 5955 | 75,9% |
| Marital status | |  | 0,0% |  |  |  |
|  | No partner | 568 | 18,1% | 482 | 1351 | 17,2% |
|  | With partner | 2571 | 81,9% | 2204 | 6493 | 82,8% |
| Previous Conization(yes) | | 45 | 1,4% | 36 | 102 | 1,3% |
| Uterine Anomaly (yes) | | 45 | 1,4% | 36 | 117 | 1,5% |
| Obstetrical history | |  |  |  |  |  |
|  | Nulliparous | 1449 | 46,2% | 1244 | 3528 | 45,0% |
|  | Parous with no previous preterm birth | 1371 | 43,7% | 1217 |  |  |
|  | Parous with at least one previous preterm birth | 318 | 10,1% | 225 | 840 | 10,7% |
|  | Previous abortion(yes) | 767 | 24,4% | 629 | 2022 | 25,8% |
| Funneling at measurement (yes) | | 105 | 3,4% | 59 | 229 | 3,0% |
| Sludge at measurement (yes) | | 92 | 2,9% | 59 | 347 | 4,6% |
|  |  |  | **Mean** |  |  | **Mean** |
| Cervical length at measurement (mm) | |  | 37.78 |  |  | 36.9 |
|  |  |  |  |  |  |  |
