# Supplementary material for: Association between cervical length and gestational age at birth in singleton pregnancies: a multicentric prospective cohort study in the Brazilian population
Source: Reprod Health. 2023 Mar 22;20:47. doi: 10.1186/s12978-022-01557-w (PMC10035243; doi:10.1186/s12978-022-01557-w)
Supplement: Supplementary file 2 — Additional file 2: Multivariatelogistic regression analysis for total and sPTB at different gestational ages. [file 12978_2022_1557_MOESM2_ESM.docx]

Additional file 2 – Multivariate logistic regression analysis for total and sPTB at different gestational ages

|  | |  | | **PTB <37** | | **sPTB <37** | |
| --- | --- | --- | --- | --- | --- | --- | --- |
|  | |  | | **ORa (CI95%)** | **p-value** | **ORa (95%CI)** | **p-value** |
| **Body-mass index (kg/m^2^)** | | | |  |  |  |  |
|  | ≤18.5 | | | 1.99 (1.05-3.78) | <0.05 |  |  |
| **No comorbidities** | | |  | 0.57 (0.46-0.71) | <0.01 |  |  |
| **Previous abortion(yes)** | | | | 1.35 (1.00-1.82) | <0.05 | 1.69 (1.08-2.64) | <0.05 |
| **CL at measurement (mm)** | | | |  |  |  |  |
|  | ≤10mm | | | 3.82 (1.12-13.06) | <0.05 | 6.71 (1.79-25.27) | <0.01 |
|  | 10 - ≤20mm | | | 3.04 (1.54-5.71) | <0.01 | 4.59 (2.12-9.94) | <0.01 |
|  | 20 - ≤25mm | | | 1.93 (1.22-3.06) | <0.01 | 2.07 (1.14-3.76) | <0.05 |
|  | 25 - ≤30mm | | | 1.80 (1.23-2.63) | <0.01 | 2.20 (1.35-3.57) | <0.01 |
| **Funneling at measurement (yes)** | | | | 1.80 (1.02-3.19) | <0.05 |  |  |
|  |  |  |  | **PTB<34** | | **sPTB <34** | |
|  |  |  |  | **ORa (95%CI)** | **p-value** | **ORa (95%CI)** | **p-value** |
| **Body-mass index (kg/m^2^)** | | | |  |  |  |  |
|  | ≤18.5 | | |  |  | 3.90 (1.33-11.46) | <0.01 |
| **No comorbidities** | | |  | 0.53 (0.32-0.87) | <0.05 | 0.43 (0.22-0.84) | <0.05 |
| **Previous abortion(yes)** | | | | 1.98 (1.20- 3.25) | <0.01 |  |  |
| **CL at measurement (mm)** | | | |  |  |  |  |
|  | ≤10mm | | | 12.36 (3.15-48.60) | <0.01 | 23.40 (4.91-111.34) | <0.01 |
|  | 10 - ≤20mm | | | 4.04 (1.70-9.60) | <0.01 | 5.82 (2.09-16.23) | <0.01 |
|  | 20 - ≤25mm | | | 2.75 (1.48-5.12) | <0.01 | 2.57 (1.10-5.99) | <0.05 |
|  |  |  |  | **PTB<30** | | **PTB<28** | |
|  |  |  |  | **ORa (95%CI)** | **p-value** | **ORa (95%CI)** | **p-value** |
| **Uterine Anomaly (yes)** | | | | 6.73 (2.14-21.17) | <0.01 | 12.80 (3.85-42.60) | <0.001 |
| **CL at measurement (mm)** | | | |  |  |  |  |
|  | ≤10mm | | | 27.37 (5.36-139.72) | <0.01 | 82.78 (10.87-630.48) | <0.01 |
|  | 10 - ≤20mm | | | 10.70 (3.38- 33.90) | <0.01 | 9.73 (2.06-45.95) | <0.01 |
