# Supplementary material for: Association between cervical length and gestational age at birth in singleton pregnancies: a multicentric prospective cohort study in the Brazilian population
Source: Reprod Health. 2023 Mar 22;20:47. doi: 10.1186/s12978-022-01557-w (PMC10035243; doi:10.1186/s12978-022-01557-w)
Supplement: Supplementary file 3 — Additional file 3: Cervicallength x PTB with adjusted OR for BMI, comorbidities, obstetrical history,funneling and sludge (Table S3.1, S3.2 and S3.3). [file 12978_2022_1557_MOESM3_ESM.docx]

Additional file 3 - Cervical length x PTB with adjusted OR for BMI, comorbidities, obstetrical history, funneling and sludge (table S3.1, S3.2 and S3.3)

Table 3.1 - Cervical length x PTB<37 adjusted OR for BMI, comorbidities, obstetrical history, funneling and sludge

| Cervical length at measurement (mm) | **Overall PTB<37 (n=453)** | | **≥37w (n=2686)** | | **ORa (CI95%)** | **p-value** |
| --- | --- | --- | --- | --- | --- | --- |
|  | **n** | **%** | **n** | **%** |  |  |
| ≤10mm | 11 | (2.43%) | 7 | (0.26%) | 4.19 (1.37 - 13.34) | 0.013 |
| 10 ≤15mm | 9 | (1.99%) | 10 | (0.37%) | 3.17 (1.1 - 8.81) | 0.028 |
| 15 ≤20mm | 15 | (3.31%) | 32 | (1.19%) | 2.09 (0.98 - 4.26) | 0.048 |
| 20 ≤25mm | 38 | (8.39%) | 106 | (3.95%) | 1.98 (1.27 - 3.04) | 0.002 |
| 25 ≤30mm | 46 | (10.2%) | 156 | (5.81%) | 1.83 (1.25 - 2.64) | 0.001 |
| > 30mm | 334 | (73.7%) | 2375 | (88.4%) |  |  |
| Data are number (%)  n total=3139 | | | | | | |

Table 3.2 - Cervical length x sPTB<37 adjusted OR for BMI, comorbidities, obstetrical history, funneling and sludge

| Cervical length at measurement (mm) | **sPTB<37 (n=223)** | | **≥37w (n=2686)** | | **ORa (CI95%)** | **p-value** |
| --- | --- | --- | --- | --- | --- | --- |
|  | **n** | **%** | **n** | **%** |  |  |
| ≤10mm | 8 | (3.59%) | 7 | (0.26%) | 6.05 (1.7 - 21.78) | 0.005 |
| 10 ≤15mm | 6 | (2.69%) | 10 | (0.37%) | 4.38 (1.28 - 13.63) | 0.013 |
| 15 ≤20mm | 9 | (4.04%) | 32 | (1.19%) | 2.22 (0.86 - 5.15) | 0.078 |
| 20 ≤25mm | 20 | (8.97%) | 106 | (3.95%) | 1.89 (1.04 - 3.27) | 0.028 |
| 25 ≤30mm | 29 | (13.0%) | 156 | (5.81%) | 2.3 (1.44 - 3.58) | <0.001 |
| > 30mm | 151 | (67.7%) | 2375 | (88.4%) |  |  |
| Data are number (%)  n total=2909 |  |  |  |  |  |  |

Table 3.3 - Cervical length x sPTB<34 adjusted OR for BMI, comorbidities, obstetrical history, funneling and sludge

| Cervical length at measurement (mm) | **sPTB<34 (n=78)** | | **≥34w (n=2976)** | | **ORa (CI95%)** | **p-value** |
| --- | --- | --- | --- | --- | --- | --- |
|  | **n** | **%** | **n** | **%** |  |  |
| ≤10mm | 7 | (8.97%) | 9 | (0.30%) | 18.39 (4.39 - 75.93) | <0.001 |
| 10 ≤15mm | 3 | (3.85%) | 13 | (0.44%) | 7.27 (1.39 - 28.5) | 0.008 |
| 15 ≤20mm | 6 | (7.69%) | 40 | (1.34%) | 4.7 (1.43 - 13.31) | 0.006 |
| 20 ≤25mm | 10 | (12.8%) | 124 | (4.17%) | 3.25 (1.4 - 6.88) | 0.004 |
| 25 ≤30mm | 7 | (8.97%) | 192 | (6.45%) | 1.56 (0.61 - 3.43) | 0.305 |
| > 30mm | 45 | (57.7%) | 2598 | (87.3%) |  |  |
| Data are number (%) |  |  |  |  |  |  |
| n total=3054 |  |  |  |  |  |  |
