# Supplementary material for: Association between cervical length and gestational age at birth in singleton pregnancies: a multicentric prospective cohort study in the Brazilian population
Source: Reprod Health. 2023 Mar 22;20:47. doi: 10.1186/s12978-022-01557-w (PMC10035243; doi:10.1186/s12978-022-01557-w)
Supplement: Supplementary file 4 — Additional file 4: TVUmeasurement of CL performance for predicting PTB. [file 12978_2022_1557_MOESM4_ESM.docx]

Additional file 4 – TVU measurement of CL performance for predicting PTB

|  | **AUC** | **Cut off** | **Sensitivity** | **Specificity** |
| --- | --- | --- | --- | --- |
| **PTB<37** | 0.598 | 31.75 | 31.3 | 84.4 |
| **sPTB<37** | 0.643 | 31.75 | 37.2 | 84.3 |
| **sPTB<34** | 0.665 | 28.05 | 41.0 | 89.1 |
| **sPTB<32** | 0.718 | 28.05 | 52.3 | 88.9 |
| **sPTB<28** | 0.820 | 26.55 | 73.7 | 91.3 |

AUC: area under the curve.
