# Supplementary figures and images for: Association between cervical length and gestational age at birth in singleton pregnancies: a multicentric prospective cohort study in the Brazilian population
Source: Reprod Health. 2023 Mar 22;20:47. doi: 10.1186/s12978-022-01557-w (PMC10035243; doi:10.1186/s12978-022-01557-w)

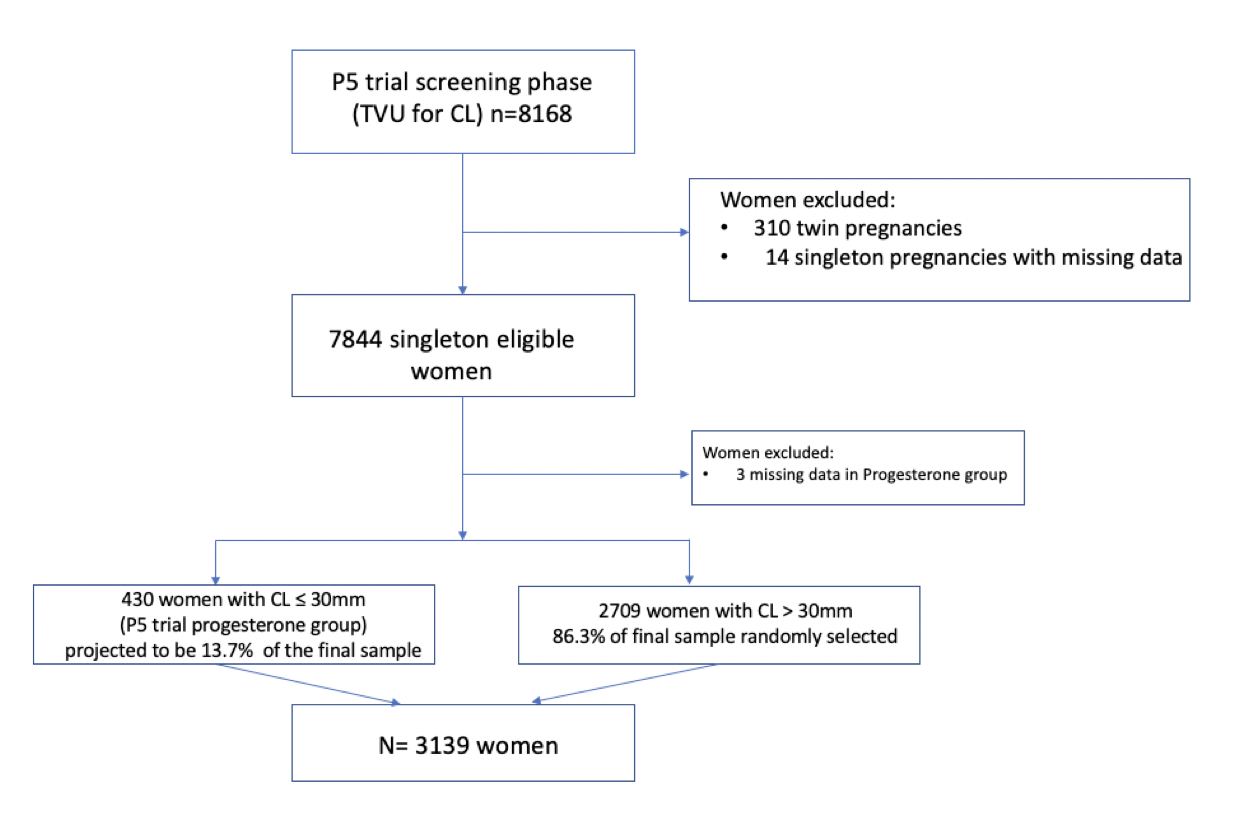

Supplement: Supplementary file 5 — Additional file 5: Women enrolment flowchart. [file 12978_2022_1557_MOESM5_ESM.tiff]

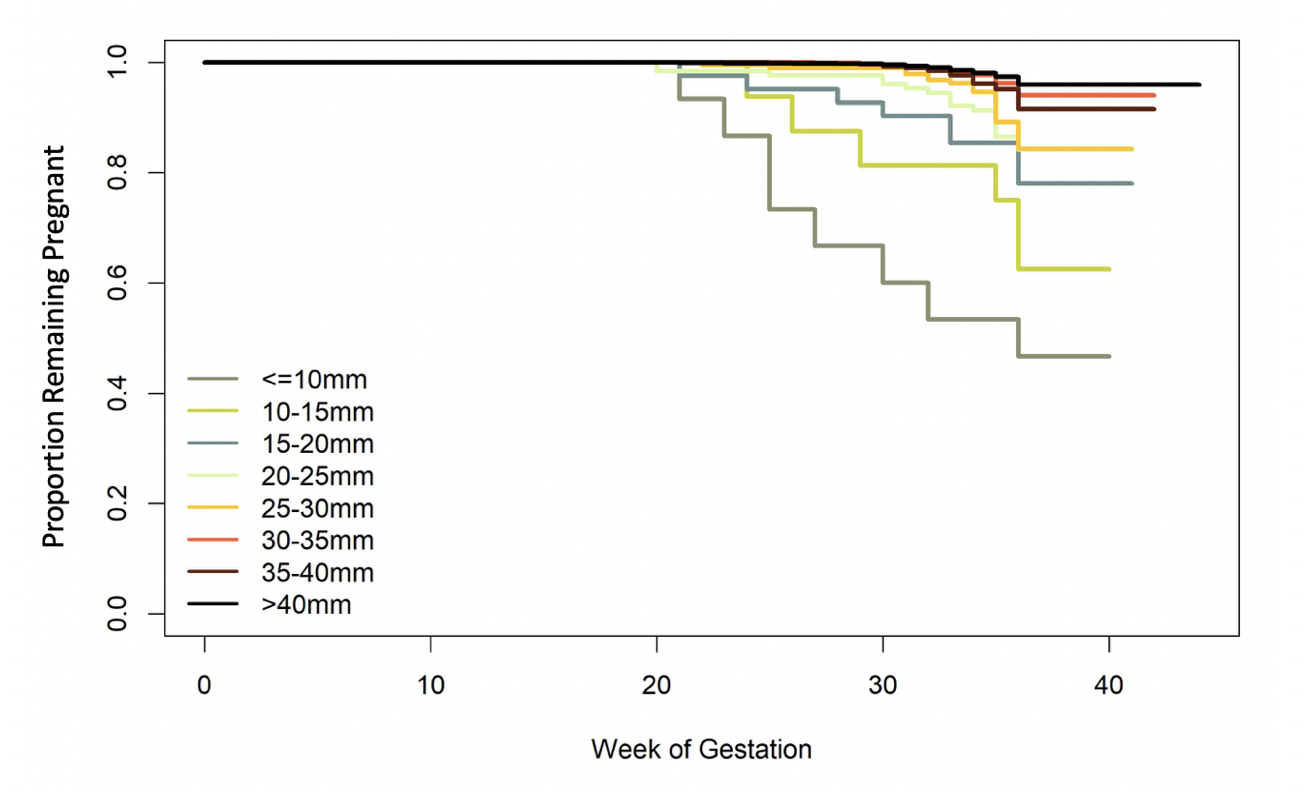

Supplement: Supplementary file 6 — Additional file 6: Kaplan-Meyer survival analysis for sPTB considering different ranges ofCL. [file 12978_2022_1557_MOESM6_ESM.tiff]
